# A longitudinal assessment of racial and ethnic inequities in food environment exposure and retail market concentration

**DOI:** 10.1017/S1368980023001179

**Published:** 2023-09

**Authors:** Qianxia Jiang, Debarchana Ghosh, Sandro Steinbach, Kristen Cooksey Stowers

**Affiliations:** 1 Center for Children’s Healthy Lifestyles and Nutrition, Children’s Mercy Kansas City, Kansas City, MO, USA; 2 Department of Geography, University of Connecticut, Storrs, CT, USA; 3 Department of Agricultural and Resource Economics, University of Connecticut, Storrs, CT, USA; 4 Department of Allied Health Sciences, University of Connecticut, Storrs, CT, USA

**Keywords:** Racial and ethnic inequities, Food environment exposure, Food retail market concentration, Longitudinal analysis

## Abstract

**Objective:**

This paper assesses trends in food environment and market concentration and racial and ethnic inequities in food environment exposure and food retail market concentration at the US census tract level from 2000 to 2019.

**Design:**

Establishment-level data from the National Establishment Time Series were used to measure food environment exposure and food retail market concentration. We linked that dataset to race, ethnicity and social vulnerability information from the American Community Survey and the Agency for Toxic Substances and Disease Registry. A geospatial hot-spot analysis was conducted to identify relatively low and high healthy food access clusters based on the modified Retail Food Environment Index (mRFEI). The associations were assessed using two-way fixed effects regression models.

**Setting:**

Census tracts spanning all US states.

**Participants:**

69 904 US census tracts.

**Results:**

The geospatial analysis revealed clear patterns of areas with high and low mRFEI values. Our empirical findings point to disparities in food environment exposure and market concentration by race. The analysis shows that Asian Americans are likelier to live in neighbourhoods with a low food environment exposure and low retail market concentration. These adverse effects are more pronounced in metro areas. The robustness analysis for the social vulnerability index confirms these results.

**Conclusion:**

US food policies must address disparities in neighbourhood food environments and foster a healthy, profitable, equitable and sustainable food system. Our findings may inform equity-oriented neighbourhood, land use and food systems planning. Identifying priority areas for investment and policy interventions is essential for equity-oriented neighbourhood planning.

Neighbourhood disparities in access to food outlets are a significant concern for the US food system^(1)^. Differences in food access can affect dietary intake and increase the risk of several diet-related negative health outcomes^(2)^. Racial and ethnic minority populations mostly living in neighbourhoods with limited access to grocery stores and other services such as healthcare and social support experience disproportionately poor health outcomes (e.g. healthy diet and physical and mental health) than their White counterparts due to systemic socio-economic inequities^(2)^.

Neighbourhoods with higher concentrations of racial and ethnic minority populations likely have food deserts and food swamps^(3,4)^. Among the several definitions of food deserts, the United States Department of Agriculture’s^(5)^ description of a food desert is the most commonly used, where US census tracts are identified as food deserts if they satisfy the following two conditions of (1) ‘*low-income communities*’, based on having a poverty rate of 20 % or greater or a median family income at or below 80 % of the area median family income and (2) ‘*low-access communities*’, based on the determination that at least 500 persons and at least 33 % of the census tract’s population live more than *one mile* from a supermarket or a large grocery store. Food swamp is defined as an area where there is an overabundance of convenience store and fast-food restaurants, swamping the surrounding areas with limited access to healthy food options^(6)^. Previous studies have shown that food access is associated with the racial composition of a neighbourhood^(7)^, with more supermarkets and grocery stores in predominantly White areas and fewer in mostly Black areas^(8)^. Cooksey *et al*., in a national study of US adults, showed that residents of food swamps report poorer dietary habits and that Black Americans are likely to live in a food swamp^(4)^. Previous research also links neighbourhoods with food swamps^(6)^ to residential segregation^(7)^, disparities in individuals’ dietary behaviours and diet-related health outcomes^(3,4)^. However, a neighbourhood’s food environment is seldom healthy or unhealthy. Instead, it is on a continuum of *healthiness* where we have food deserts and food swamps on the one end and food oases (*areas with an abundance of supermarkets and a variety of grocery stores*) on the other^(9)^. A recent study measured food exposure based on perceived measures of a food desert and food swamp^(4)^.

The market structure of US food retailing is changing rapidly, both in rural and urban areas^(10)^. This trend has significantly impacted retail competition and, more specifically, consumer choices for healthy food; as a result of such structural changes in the retail market over the past decades, how and where people purchase food has changed^(10)^. For example, several empirical studies show that concentration in the food retail market may influence access to grocery stores and supermarkets and pricing^(11,12)^. For instance, Ma *et al*. show that supermarkets do not raise prices in local markets in response to firm market shares.^(11)^ Instead, smaller food retailers charge substantially higher prices on average than supermarkets. Another study has shown associations between evolving food retail landscape with inequities in access to healthy food, leading to health disparities related to poor diet and diet-related health outcomes^(13)^. The studies, however, either perceive food access primarily from economic outcomes^(14)^ or for the general population, missing the critical associations of racial and ethnic inequities in food access. However, few studies have explored the racial and ethnic equities in combining food environment exposure and food market concentration at the neighbourhood level over time. Such insights are crucial for food system planners to identify racial and ethnic minority neighbourhoods disproportionately burdened by inequitable access to healthy food and often have unhealthy food retail environments^(15)^.

In this study, we conduct a longitudinal investigation of racial and ethnic inequities in food environment exposure across the USA. Using establishment-level food retail data from the National Establishment Time Series (NETS) dataset from Walls & Associates^(16)^ for 2000–2019, we assess food environment exposure and market concentration at the census tract level. Although the NETS dataset was used in large-scale studies^(14)^ focused on food markets and measuring food swamps before, to the best of our knowledge, this is the first longitudinal study that will reveal the associations between race and ethnicity and food environment exposure and retail food market concentration. In addition, the longitudinal dataset will enable controlling for unobserved characteristics at the census tract level using two-way fixed effects regression models.

## Methodology

We used US census tracts (*n* 69 904) as the neighbourhood unit of analysis. Typically encompassing 2500–8000 people, a census tract is smaller than a city but larger than a block group or census block.^(17)^ The census tract is equivalent to a neighbourhood established by the Bureau of Census for analysing stable population characteristics and is typically integrated with extensive national surveys at the individual level^(18)^.

### Food retail market concentration measurement

The food retail market concentration was constructed from NETS, a longitudinal dataset compiled from Dun & Bradstreet archival establishment information. NETS contains longitudinal data from 1990 on various dynamics of the US economy, such as establishment job creation and destruction, sales growth performance, survivability of business start-ups, mobility patterns and changes in primary markets^(16)^. To align with the years that publicly available US census data on racial and ethnic characteristics are available, we used NETS data from 2000 to 2019. Each retail establishment can be tracked over time and classified based on the North American Industry Classification System (NAICS). NAICS is a classification of business establishments by the type of economic activity. Each food retail establishment’s location information (geographic coordinates) was extracted from NETS, geocoded and then aggregated at the census tract level using the Python GeoPandas library. Geocoding is the process of converting addresses into geographic coordinates used to place markers on a map. We used the Herfindahl–Hirschman index (HHI) to measure the food retail market concentration^(19)^. The index is calculated by squaring the market share of each firm competing in a census tract and then summing the resulting numbers. A market with an HHI of less than 1500 is considered competitive, an HHI of 1500–2500 is moderately concentrated and an HHI of 2500 or greater is highly concentrated. The Department of Justice considers highly concentrated markets, according to the HHI, as markets where companies have considerable market power^(20)^. Such market power leads to anticompetitive behaviour and often causes the Federal Trade Commission to intervene in merger and acquisition cases following the Federal Horizontal Merger Guidelines^(20)^.

### Measuring food environment exposure

Following previous research^(4,6,21)^, we categorised food retailers into seven categories using the following NAICS codes: supermarkets/grocery stores (445 110), fruit and vegetables markets (445 230), supercentres (452 311), convenience stores (445 120), dollar stores (452 319), full-service restaurants (722 511) and limited-service restaurants (722 513). A detailed description of retail formats is provided in Appendix 1. The food retail data from NETS need to differentiate between smaller and independently owned grocery stores. Previous studies have shown that many smaller stores are considered corner stores^(22)^. Therefore, we identified these stores as convenience stores if they had a NAICS code designation for a grocery store and fewer than five employees^(22)^. Dollar stores are categorised as general merchandise stores in NAICS code 452319. To incorporate dollar stores exclusively, we followed the previous research^(23)^ and restricted our sample to establishments with ‘dollar’ in the establishment name.

Consistent with previous studies on food swamps, each food retail outlet mentioned above was categorised into unhealthy, intermediate and healthy^(24)^. The unhealthy category included convenience stores and limited-service restaurants. Full-service restaurants were in the intermediate food retail outlet category drawing from prior studies that found no association between full-service restaurant access and the risk of obesity^(25)^. Supermarkets/grocery stores, fruit and vegetable markets and supercentres are in the healthy retail food outlet category. Various associations between these food outlets and dietary and health outcomes are documented in the literature^(25,26)^. We computed the modified Retail Food Environment Index (mRFEI) at the census tract level using these food retailers. The mRFEI is a score measuring the relative healthiness of a food environment, a continuum where we have limited access to healthy food (food desert) or higher availability of unhealthy food (food swamp) on the one end and higher availability and easy access to healthy food (food oasis) on the other end. The mRFEI is calculated as follows^(6)^:
(1)

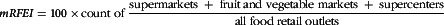




### Explanatory and control variables

We used racial (i.e. White, Black and Asian) and ethnicity (i.e. Hispanic) proportions at the census tract level as the primary explanatory variables. These variables for 2000 and 2010 were obtained from the Decennial Census and 2019 from the 2015–2019 5-year estimate American Community Survey sample data. Several other factors may influence food environment exposure and market concentration. In line with previous research, we included the following control variables at the tract level: the overall population size (the log of the population)^(7)^; educational attainment (the percentage of people having a Bachelor’s degree or higher)^(16)^ and the poverty rate^(27)^. The above control variables were also obtained from the Decennial Census (2000 and 2010) and the American Community (2015–2019). The metropolitan and non-metropolitan classification of the census tract is based on the 2013 United States Department of Agriculture Rural-Urban Continuum Codes shown in Appendix 2 ^(28)^.

Moreover, we used the social vulnerability index (SVI) as a robustness check. The SVI indicates the potential negative effects of US census tract areas^(29)^, which includes an overall score and four sub-theme scores (i.e. socio-economic status, household composition and disability, minority status and language, and housing type and transportation). The overall SVI census tract rating was calculated based on four sub-themes. We used the overall census tract rating and four summary sub-themes from 2000 to 2019. Appendix 3 provides further detail, and Appendix 4 shows the descriptive statistics.

### Analytical approach

#### Statistical analysis

We used two-way fixed effects regression models to investigate racial and ethnic inequities in the food environment^(30)^. The baseline model is specified as follows:
(2)

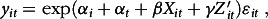




where we denote the census tract with 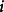 and the year with 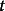 . The outcomes of interest ( 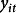 ) are the mRFEI and HHI. Because the data are left-skewed, we use an exponential regression model to identify the parameters of interest. We control for unobserved time-invariant factors with census-tract fixed effects ( 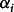 ) and account for common shocks over time with time fixed effects ( 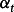 ). We balanced the dataset at the census tract level and kept census tracts with zero outcomes available from 2000 to 2019. The variable of interest *X_it_* represents the racial and ethnic composition (%) within a given census tract and year. The statistical model identifies the association between the outcome and treatment variables through variation within census tracts and over time. We denote the set of control variables with 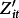 , and the multiplicate error term with 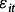 . After dropping observations that were either singletons or separated by a fixed effect, 39 965 (mRFEI model) and 47 486 (HHI model) census tracts were included in the regression analysis.

We follow the standard practice of dealing with abundant zeros and rely on the Poisson pseudo maximum likelihood estimator to identify the parameters of interest in Equation 1^(31,32)^. Silva and Tenreyro showed that Poisson pseudo maximum likelihood is robust to different patterns of heteroskedasticity and measurement error^(33)^. The estimator also allows us to address the large share of zeros in the dataset consistently. We account for the high-dimensional fixed effects using a modified version of the iteratively re-weighted least-squares algorithm robust to statistical separation and convergence issues^(34)^. Lastly, we suspect that the standard errors are correlated within census tracts, prompting us to cluster them at this level^(35)^.

#### Geospatial analysis

Geospatial analyses of food environments, including supermarkets, convenience stores, restaurants or fast-food centres, and their impact on dietary health have been commonly used over the past 25 years ^(36)^. As spatial data and geographic information systems (GIS) software became widely available to researchers and practitioners across the social sciences, public health and medicine, mapping and geospatial analysis have been the predominant methods. One review published in 2012 found that 53 % of published research on the food environment used geospatial analysis ^(37)^. Another systematic review from 2017 found that 49·6 % of articles focused on food access included geospatial analysis before 2007; from 2007 through 2015, that percentage increased to 65·3 %^(38)^.

Following this trend, we used cluster analysis in a GIS framework to identify statistically significant clusters of census tracts with low and high mRFEI values. Low mRFEI indicates limited access to healthy food options, while a high mRFEI suggests greater access to healthy food choice.

First, we prepared the GIS files by merging the boundary files of the census tracts^(39)^ with mRFEI score and the racial (i.e. White, Black and Asian) and ethnicity (i.e. Hispanic) proportions.

Second, we conducted a hot-spot analysis using ESRI’s ArcGIS Pro 2.8 GIS software^(40)^. We implemented this analysis for 2000, 2010 and 2019. Based on a set of weighted features (here, mRFEI scores), the hot-spot analysis function identifies statistically significant hot spots and cold spots using the Getis-Ord Gi* statistic^(41)^. Hot spots are regions with higher mRFEI scores indicating better access to healthy foods or food oases. Cold spots are regions with lower mRFEI scores indicating higher relative access to unhealthy food retailers^(42)^, showing likely situations of food deserts or food swamps. The hot-spot analysis tool creates an output with a z-score, *P*-value and confidence levels for each census tract. The z-scores and *P*-values are measures of statistical significance that tell us whether to reject the null hypothesis of no clustering pattern tract by tract. In effect, they indicate whether the observed geographical clustering of high or low values is more pronounced than expected in a random distribution of those same values. A high z-score and small *P*-value for a census tract indicate a spatial clustering of high mRFEI values or food oasis. A low negative z-score and small *P*-value indicate a spatial clustering of low mRFEI values, indicating food swamps or deserts. We use three confidence intervals for the identified clusters for statistical significance: 99 %, 95 % and 90 %. We want to briefly note that hot-spot analysis of HHI value (i.e. identifying areas with high and low concentrations of retail markets) did not provide meaningful results, perhaps due to large numbers of census tracts with null or zero HHI values.

## Results

### Temporal trends in food environment exposure and retail market concentration

**Fig. 1:**
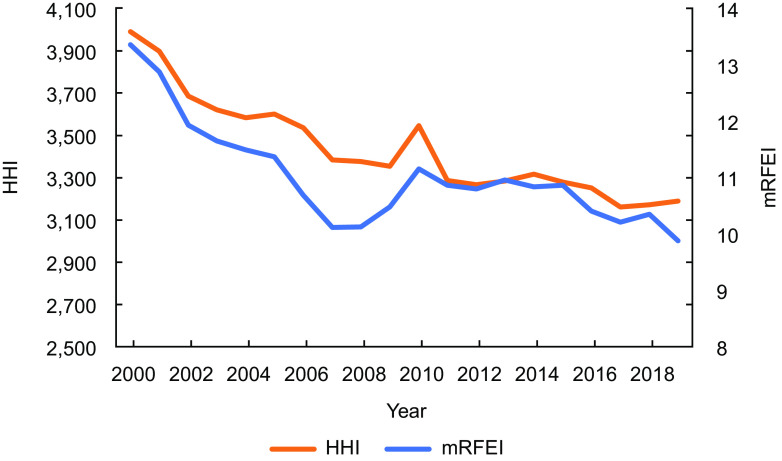
Trends in food environment exposure and market concentration 2000–2019

Figure 1 shows trends in food environment exposure and market concentration from 2000 to 2019. On average, we find that food environment exposure decreased slightly (mRFEI decreased to 26 % in 2019 compared to 2000), while market concentration also reduced during the study period (HHI decreased 20·1 % in 2019 compared to 2000).

### Statistical analysis results

Table 1 summarises the baseline results for the associations between the racial and ethnic proportions and food environment exposure measure, that is, mRFEI. We found that the proportion of White people at the census tract level is positively associated with the mRFEI. In contrast, the proportion of Asian people was negatively associated with the mRFEI, suggesting that census tracts with more White people experience healthier food environments. The opposite relationship holds for the proportion of Asian people. The ratio of White, Asian and Hispanic at the census tract level was negatively associated with HHI, suggesting that census tracts with more White, Asian and Hispanic people were associated with more competitive markets.

**Table 1:** Food environment and racial and ethnic proportions

	Food environment exposure		Market concentration	
	*β*	sig.	*β*	Sig.
White proportion	0·00128	0·006	−0·00066	0·003
Black proportion	−0·00038	0·586	−0·00046	0·129
Asian proportion	−0·00281	0·008	−0·00531	< 0·001
Hispanic proportion*	0·00065	0·265	−0·00156	< 0·001
Poverty percentage	0·00003	0·700	−0·00012	0·002
Bachelor or higher percentage	0·00032	0·001	0·00007	0·083
Population	−0·11 987	< 0·001	−0·17 633	< 0·001

Food environment exposure: Number of observations = 798 500; residual df = 39 965. R squared = 0·46.

Market concentration: Number of observations = 948 571; residual df = 47 486. R squared = 0·74.

* The impact of the Hispanic proportion was assessed in a separate model with the same outcome and control variables.

Table 2 assesses the associations for the baseline model separately for metro and non-metro areas. We found that the White and Hispanic proportions were positively associated with mRFEI for metro census tracts. In contrast, the Black ratio was negatively associated with mRFEI for non-metro tracts. This result aligns with the geospatial finding that the size or the geographic coverage of the cold spots of mRFEI scores indicating limited access to healthy foods increased over the years from the selected metros to its suburbs. In addition, we found that White and Asian proportions were negatively associated with HHI in metro areas for market concentration. However, only the White ratio was negatively associated with HHI among non-metro census tract areas.

**Table 2:** Food environment and racial and ethnic proportions for metro *v*. non-metro areas

	Food environment exposure				Market concentration			
	Metro		Non-metro		Metro		Non-metro	
	*β*	Sig.	*β*	Sig.	*β*	Sig.	*β*	Sig.
White proportion	0·00158	0·002	−0·00075	0·504	−0·00099	< 0·001	0·00172	0·003
Black proportion	0·00019	0·803	−0·00353	0·040	−0·00038	0·228	0·00095	0·256
Asian proportion	−0·00195	0·083	−0·00281	0·478	−0·00421	< 0·001	−0·00176	0·319
Hispanic proportion*	0·00136	0·027	−0·00250	0·145	−0·00011	0·713	−0·00129	0·133
Poverty percentage	−0·00002	0·828	00026	0·095	−0·00004	0·307	−0·00021	0·014
Bachelor or higher percentage	0·00033	0·002	0·00016	0·499	0·00002	0·600	0·00006	0·621
Population	−0·09667	< 0·001	−0·21 248	0·001	−0·15 471	< 0·001	−0·18 526	< 0·001

Food environment exposure (metro): Number of observations = 671 745; residual df = 33 627. R squared = 0·46.

Food environment exposure (non-metro): Number of observations = 126 755; residual df = 6338. R squared = 0·46.

Market concentration (metro): Number of observations = 804 657; residual df = 40 291. R squared = 0·75.

Market concentration (non-metro): Number of observations = 143 113; residual df = 7194. R squared = 0·75.

* The Hispanic proportion was assessed in a separate model with the same outcome and control variables.

Table 3 investigates the robustness of our baseline results using SVI scores as the explanatory variables. These estimates indicate that minority status and language sub-theme scores were negatively associated with mRFEI, suggesting that the greater the diversity of minority status and language, the unhealthier the food environment. Furthermore, the sub-theme scores for socio-economic status were negatively associated with HHI, suggesting that the greater the vulnerability of socio-economic status and minority status and language, the higher the market concentration in each census tract. At the same time, we found that the household composition and disability score were positively associated with HHI, and the relationships between the total SVI score and mRFEI and HHI were not statistically significant.

**Table 3:** Food environment and the social vulnerability index

	Food environment exposure		Market concentration	
	*β*	Sig.	*β*	Sig.
Model 1				
SVI total score	−0·012	0·690	−0·018	0·172
Model 2				
Socio-economic status	0·009	0·740	−0·039	0·001
Household composition and disability	−0·017	0·333	0·096	< 0·001
Minority status and language	−0·042	0·034	−0·112	< 0·001
Housing type and transportation	0·013	0·513	−0·016	0·070

Food environment exposure (model 1): Number of observations = 871 871; residual df = 52 866. R squared = 0·12.

Food environment exposure (model 2): Number of observations = 871 871; residual df = 52 866. R squared = 0·12.

Market concentration (model 1): Number of observations = 1 060 563; residual df = 65 264. R squared = 0·09.

Market concentration (model 2): Number of observations = 1 060 563; residual df = 65 264. R squared = 0·09.

### Geospatial analysis results

**Fig. 2:**
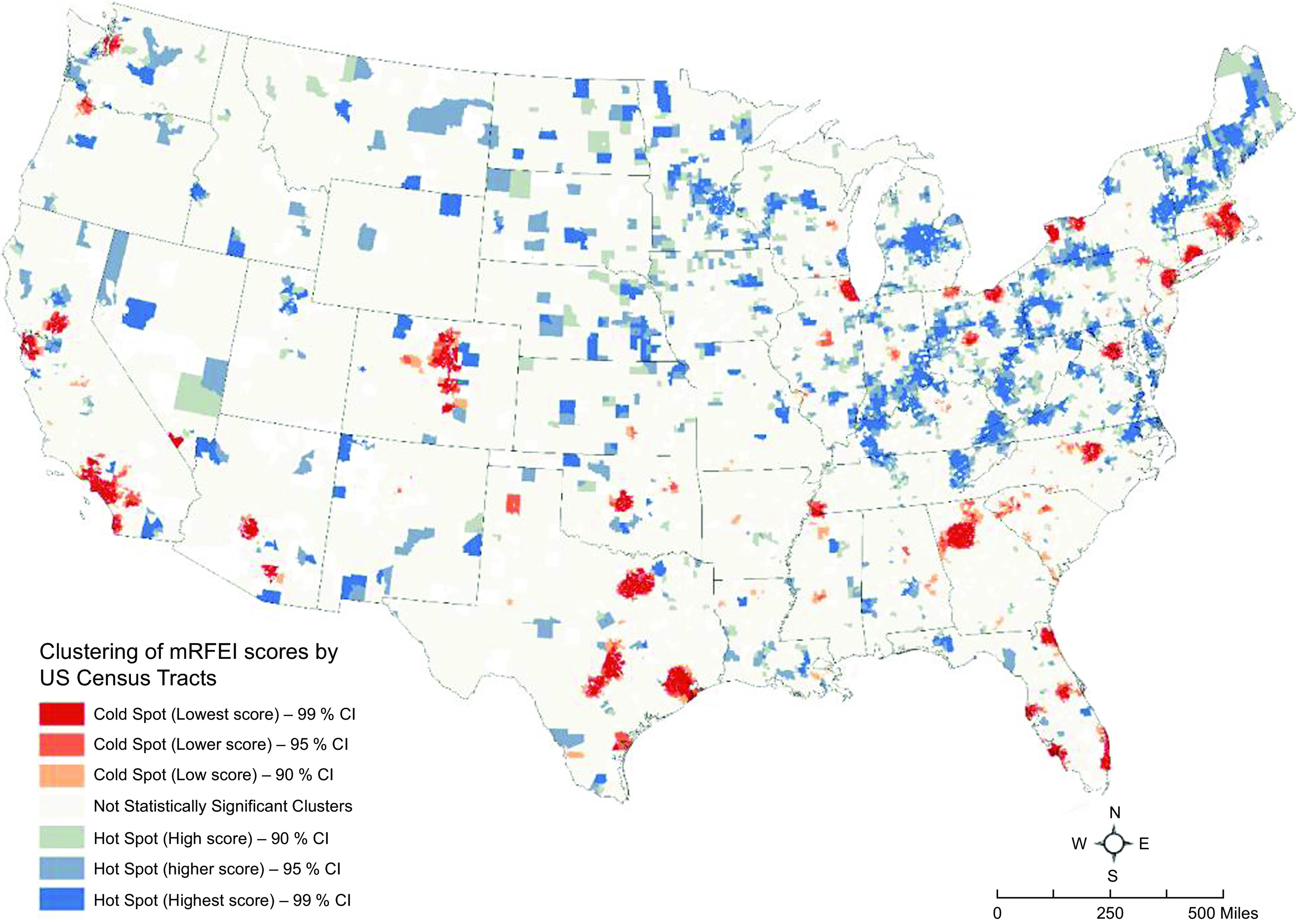
Hotspot analysis of modified Retail Food Environment Index mRFEI scores in 2000 (tracts in the lower 48 US states)

**Fig. 3:**
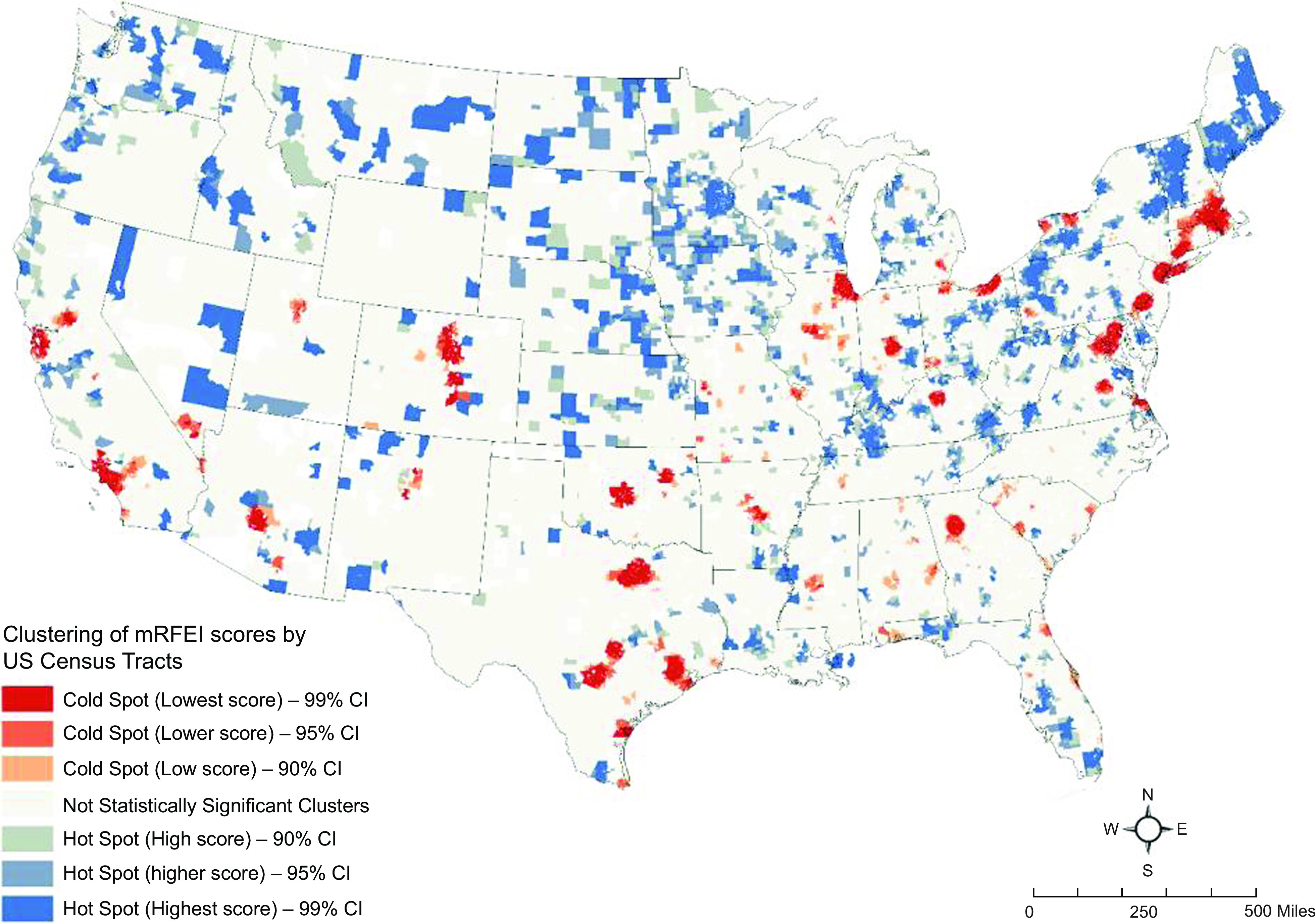
Hotspot analysis of modified Retail Food Environment Index (mRFEI) scores in 2010 (tracts in the lower 48 US states)

**Fig. 4:**
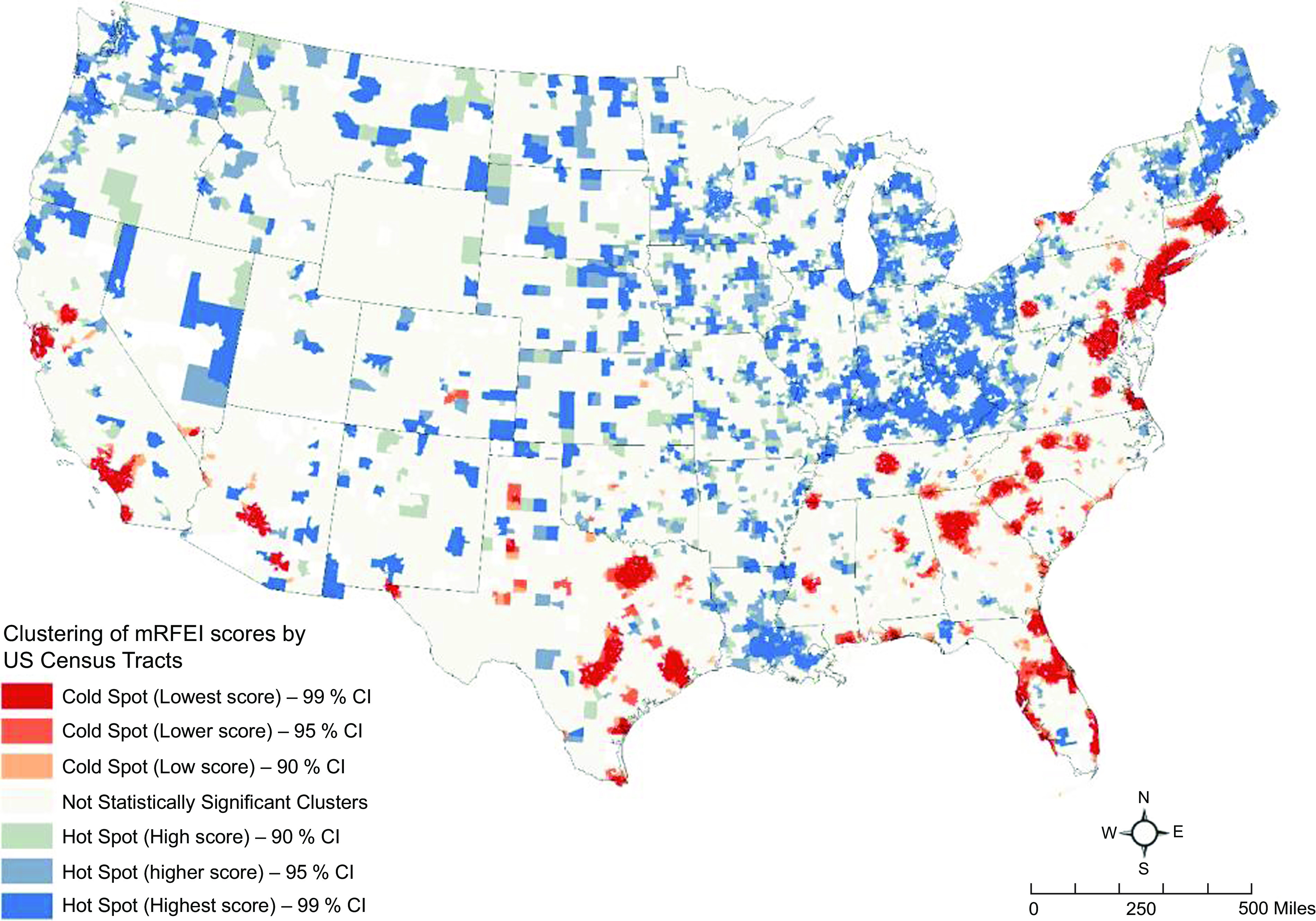
Hotspot analysis of modified Retail Food Environment Index (mRFEI) scores in 2019 (tracts in 48 US states)

Figures 2, 3 and 4 show the results of the geospatial analysis that identifies statistically significant hot and cold spots of mRFEI scores in 2000, 2010 and 2019, respectively. Hot spots, that is, clusters of census tracts (in shades of blue) with higher mRFEI scores, indicate neighbourhoods with higher availability and easy access to healthy food retailers. Hot spots can be related to food oases. Conversely, cold spots, that is, clusters of census tracts (in shades of red) with lower mRFEI scores, indicate neighbourhoods with lower availability (or higher availability of unhealthy foods) and limited access to healthy food retailers. Cold spots can be related to food deserts or food swamps.

Several spatial patterns of mRFEI scores were observed during the study period. First, the number of statistically significant clusters, including hot and cold spots, increased from 2000 to 2019, with the number of hot spots (higher mRFEI scores; easy access to healthy food) growing more than cold spots. Second, in some cold spots, that is, areas with limited access to healthy food (a possible situation of food deserts) or higher availability of unhealthy *v*. healthy foods (a possible case of food swamps), accessibility to healthy food improved over time, and they changed to hot spots. Some examples include census tracts in cities in Illinois (e.g. Chicago), Ohio (e.g. Cleveland, Columbus) and Colorado (e.g. Denver) (see Figs 2 and 4). Third, even though no new cold spot emerged over time, the area of the existing cold spots increased spatially (i.e. the spot began wider or longer), indicating more inequity (or segregation) in the relative access to unhealthy compared to healthy foods. Such clusters are in Texas, Florida, the southern states, including Alabama, Georgia and South Carolina, and the northeastern states, including New Jersey, New York, Connecticut and Massachusetts (Fig. 4). Fourth, the spatiotemporal pattern of the hot and cold spots of mRFEI scores or food environment exposure in the western states did not change significantly.

### Conclusion

This paper provides the first systematic assessment of racial and ethnic inequities in food environment exposure and market concentration at the census tract level for the USA over the past two decades. According to the descriptive results, food swamps expanded from 2000 to 2019 in areas where food retail outlets with limited healthy food (and more unhealthy food) choices grew substantially while independent full-service grocery or supermarket stores fell behind, creating a situation of supermarket redlining^(2)^. The term supermarket redlining describes the disinclination of chain supermarkets to locate or relocate existing stores from impoverished inner-city neighbourhoods to affluent suburbs. As with more familiar forms of banking and residential redlining, the driving force behind supermarket redlining is also an abstraction based on perceived ‘urban obstacles’, including racial segregation^(43)^. Supermarkets also tend to drive smaller local grocery stores out of business when they move in. When they relocate or close, residents face difficulties in accessing healthy and affordable food, thus widening the grocery gap, increasing food insecurity and unhealthiness of the food environment. In a case study of the city of Hartford^(2)^, with a higher proportion of Black American and low-income communities, mapped critical areas in the inner-city region where if a nearby supermarket closes or relocates to a suburb with little mitigation efforts to fill the grocery gap, a large number of minority, poor and disadvantaged residents will experience difficulties to access healthy food. This is of concern as earlier studies showed that unhealthy food environment such as food swamp strongly predicts geographic disparities in adult obesity prevalence^(6)^.

We found strong evidence for racial and ethnic inequities in food environment exposure at the census tract level. Our regression model with the SVI also indicated that the greater diversity of minority status and language was associated with an unhealthier food environment. We also found that these racial and ethnic inequities in food environments differed among the metro and non-metro areas. Some of our findings align with previous studies on structural racism in the food system^(2,44)^. These studies found that racial and ethnic minorities are likelier to live near unhealthy food retail outlets than White. For example, Black and Hispanic people tended to have greater access to fast-food establishments than their white counterparts^(44)^. Previous research also pointed to racial and ethnic disparities in the likelihood of residents living in a food swamp or desert^(4)^. Additional research examining structural racism and residential segregation as a fundamental cause^(45)^ of health inequities is needed, particularly as its relates to the clustering of health restricting resources (e.g. unhealthy food outlets). Additional research on these topics is warranted to better inform federal nutrition policies, neighbourhood revitalisation efforts, land use zoning and housing policies to combat racial residential segregation.

Except for minority status and language score, we found no significant correlations between the social vulnerability sub-theme scores (i.e. socio-economic status, household composition and disability, housing type and transportation). The previous literature suggested that vacant homes may influence the composition of food outlets in urban neighbourhoods^(46)^. Specifically, a study found that an increase in the vacancy home rate was negatively associated with the food swamp index from 2001 to 2012, in Baltimore, among non-African American neighbourhoods. Furthermore, research also showed that neighbourhood food access varies significantly with a neighbourhood’s socio-economic composition^(47)^. However, we did not detect evidence for these associations at the national level. Therefore, a more comprehensive understanding of the specific roles of housing type and socio-economic composition in shaping the food environment may be helpful for policymakers, city planners and public health practitioners to promote healthy food access among neighbourhoods.

The current study does have a few limitations. First, the secondary data source we used to measure the local food environment may need to accurately explore the associations we found for some study areas because we cannot observe the product range offered by the stores. Realistically, food environment field audits cannot measure food establishments in a large region or during a longer historical period because such efforts are generally cost-prohibitive^(48)^. Previous studies suggest that InfoUSA and government food registries have a higher level of agreement than reported by other secondary data sources^(49)^. Future work might improve consistency in data gathering, geocoding, editing and analysing secondary data sources. Previous evidence also showed that data providers might apply classification schemes inconsistently^(50)^. Lastly, our study could have undercounted the number of dollar stores as there is no specific NAICS code for this retail outlet type. Further research may involve within-store measures of previous menu audit studies to classify different formats of retail food outlets^(18)^.

Our study findings, nevertheless, may be used to identify racial and ethnic minority neighbourhoods burdened by unhealthiness of food environments with food deserts and food swamps and to inform equity-oriented food systems and neighbourhood planning. In addition, initiatives aiming to simultaneously incentivise additional healthy food retailers (e.g. Healthy Food Financing Initiative, which provides subsidies for supermarket operators that locate in food deserts) and disincentivise unhealthy food retail environments (e.g. zoning restrictions, stocking standards for SNAP-authorised corner stores) could play a crucial role in ensuring equitable access to healthy food, particularly in communities of colour. Our findings suggest that US food policies and economic incentives must foster a healthy, fair and sustainable food system to address disparities in neighbourhood healthy food environments. Finally, future research may explore how citizenship and immigrant status affects widening food environment disparities between Hispanic populations and other people of colour.

## Appendix 1 Definitions of Retail Format

**Table:**

Format	NAICS code	Definition
Supermarkets/grocery stores	445110	The industry comprises establishments generally known as supermarkets and grocery stores primarily engaged in retailing a general line of food, such as canned and frozen foods; fresh fruits and vegetables; and fresh and prepared meats, fish and poultry
Fruit and vegetables markets	445230	This industry comprises establishments primarily engaged in retailing fresh fruits and vegetables
Supercentres and warehouse clubs	452311	This industry comprises establishments known as warehouse clubs, superstores or supercentres primarily engaged in retailing a general line of groceries in combination with general lines of new merchandise, such as apparel, furniture and appliances
Convenience stores	445120 and those with employees four or less in NAICS code 445110	This industry comprises establishments known as convenience stores or food marts primarily engaged in retailing a limited line of goods that generally includes milk, bread, soda and snacks
Dollar stores	452319	Stores with ‘dollar’ in the name with NACIS code 452319
Full-service restaurants	722511	This industry comprises establishments primarily engaged in providing food services to patrons who order and are served while seated (i.e. waiter/waitress service) and pay after eating
Limited-service restaurants	722513	This industry comprises establishments primarily engaged in providing food services where patrons generally order or select items and pay before eating. Food and drink may be consumed on premises, taken out or delivered to the customer’s location

Sourced from the North American Industry Classification System code definitions.

## Appendix 2 Categories for metro and non-metro counties

**Table:**

Code	Description
Metro counties	
1	Counties in metro areas of 1 million population or more
2	Counties in metro areas of 250 000–1 million population
3	Counties in metro areas of fewer than 25 000 population
Non-metro counties	
4	Urban population of 20 000 or more, adjacent to a metro area
5	Urban population of 20 000 or more, not adjacent to a metro area
6	Urban population of 2500–19 999, adjacent to a metro area
7	Urban population of 2500–19 999, adjacent to a metro area
8	Completely rural or less than 2500 urban population, adjacent to a metro area
9	Completely rural or less than 2500 urban population, not adjacent to a metro area

## Appendix 3 Components of the social vulnerability index^(29)^

**Table:**

Overall vulnerability	Sub themes	Components
Social vulnerability	Socio-economic status	Below poverty
		Unemployed
		Income
		No high school diploma
	Household composition and disability	Aged 65 or older
		Aged 17 or younger
		Civilian with a disability
		Single-parent households
	Minority status and language	Minority
		Speaks English ‘less than well’
	Housing type and transportation	Multi-unit structures
		Mobile homes
		Crowding
		No vehicle
		Group quarters

## Appendix 4 Summary statistics

**Table:**

Variable	Mean	sd	Min	Max
Population	4338·89	1880·40	1	59 947
White proportion	72·90	26·14	0	100
Black proportion	14·12	23·15	0	100
Asian proportion	4·30	8·59	0	96
Hispanic proportion	14·76	21·21	0	100
Poverty percentage	15·74	22·24	0	100
Bachelor or higher percentage	17·54	22·78	0	100
HHI	3438·79	2262·78	87·96	10 000
mRFEI	11·02	14·37	0	100
SVI total score	0·511	0·284	0	1
Socio-economic status	0·505	0·283	0	1
Household composition and disability	0·507	0·278	0	1
Minority status and language	0·504	0·280	0	1
Housing type and transportation	0·517	0·276	0	1
